# Application of DSP2 for biological sex estimation in a Spanish sample: analysis based on sex and side

**DOI:** 10.1007/s00414-024-03358-1

**Published:** 2024-11-15

**Authors:** Marta San-Millán, Varsha Warrier, Anna Carrera, Francisco Reina

**Affiliations:** 1 Medical Sciences Department, Clinical Anatomy, Embryology and Neuroscience Research Group (NEOMA), Faculty of Medicine, University of Girona, Girona, Spain; 2 School of Sciences, College of Science and Engineering, University of Derby, Derby, UK

**Keywords:** Forensic anthropology, Sex estimation, Pelvis, Innominate, *Os coxa*, DSP, DSP2

## Abstract

Applying existing sexing methodologies to different populations, and reporting these findings is important to enhance their applicability and accuracy in real cases across the world. DSP was published in 2005 (Murail et al., 2005) and updated as a DSP2 in 2017 (Brůžek et al., 2017) based on a database of 10 pelvic measurements from 2040 individuals worldwide. These tools have been applied subsequently to various populations, however, its applicability to a dry Spanish population is lacking. 303 hipbones belonging to 157 individuals from the School of Legal Medicine from the University Complutense of Madrid (Spain), of which 140 individuals were documented, were analyzed to investigate the reliability, applicability and accuracy of the DSP2 sex estimation methodology, examining side and sex-based potential differences for the first time. In most of the DSP variables, intra-rater reliability showed excellent results and % applicability was higher than 85.0%. Overall % accuracy was higher than 94.0% regardless of the number or discriminant power of the utilized DSP variables. However, % sexing decreased when less variables or less discriminant ones were used for estimations, reaching 45.51% (left) and 43.31% (right). Regarding sexual dimorphism, females’ results of % applicability, % sexing and % accuracy were higher compared to males. In addition, left *os coxae* achieved better outcomes (aforementioned percentages) in most of the cases in the sex-pooled sample. Decreasing the mandatory posterior probability by 10% yielded an increase in the % sexing but reduced % accuracy, and thus, does not seem to enhance the approach’s performance. The present study validates the applicability and reliability of DSP for sexing a Spanish population. Future investigations will attempt to assess its applicability within virtual anthropology.

**Supplementary Information:**

The online version contains supplementary material available at 10.1007/s00414-024-03358-1.

## Introduction

Estimating the sex of adult skeletal remains is a critical step in the reconstruction of anthropological profiles, and by extension, human identification, particularly in medico-legal or osteo-archaeological contexts. Despite numerous anatomical elements within the skeletal framework that have been utilised for deriving sexing models [1], the skull and pelvis have demonstrated a greater predominance for sex estimation across literature [2–5]. Between these two markers, the direct relationship of the pelvis with biological sex, attributable to its active role in parturition, renders pelvic characteristics more accurate for sexing, in comparison to cranial parameters [6]. Although some enriching approaches have exhaustively described sexing variables using graphic explanations and introduced greater objectivity through score-based approaches [7], these visual or morphognostic techniques, compared to metric ones, continue to remain problematic. Visual techniques, in addition to generally being highly subjective, which results in higher intra-inter-observer errors, warrant prior experience to be able to apply them accurately [7]. Morphometric techniques, on the other hand, are more objective, repeatable, and verifiable, which have led researchers to prefer them over the former [8, 9].

Murail and colleagues developed a new morphometric technique (DSP: *Diagnose Sexuelle Probabiliste* = Probabilistic Sex Diagnosis) in 2005 [10] to estimate the sex of skeletal remains using ten pelvic measurements and obtained an overall accuracy value of 99.63%. This methodology was originally created based on a worldwide reference sample of 2040 individuals from Europe (France, England, Portugal and Lithuania), Africa (South Africa), North America (United States of America) and Asia (Thailand). In addition to this geographical diversity, different ethnic groups (Zulu, Soto, Afrikaner, African American, and European American) and temporal periods (from 18th to late 20th centuries) were also considered within the analyses to reinforce its potential applicability in miscellaneous populations. In 2017, a freely available updated software, the DSP2, was created and validated using two new samples from the Maxwell Museum Documented Collection (University of New Mexico, Albuquerque, United States) and the Simon Collection of identified skeletons (Department of Anthropology, University of Geneva, Switzerland) [11].

Since its publication, this pelvic-based ten-variable metric methodology has been tested in geographically diverse, documented collections with accuracy outcomes ranging from 88.34 to 100%: France [12], Greece [13], Mexico [14] and Brazil [15–17]. This approach has also shown high reliability and accurate results with virtual/ digital samples, i.e., using CT images from Europe, specifically Belgium [18], France [19], and Denmark [20]. Furthermore, these proven advantages led to its application to past population samples from Neanderthals [21], Pre-Columbian mummies [22], Gravettian individuals [23], Joseon Dynasty Koreans [24] and medieval skeletal remains [25]. Although the original studies advocated for the existence of a common sexual pattern worldwide [10, 11], to the best of our knowledge, this is the first time the DSP approach is being tested in a Spanish documented collection.

This study aimed to (1) analyze the intra-rater reliability, applicability, and accuracy of the DSP2 method when applied to a Spanish documented collection for sex estimation; (2) examine and compare the % applicability, % sexing and % accuracy of DSP2 among sexes and sides; (3) investigate how the combination of utilized DSP2 variables and the posterior probability can change the accuracy outcomes and (4) compare the sexing and accuracy percentages reported across different populations.

## Materials and methods

### Sample

The sample used for this investigation is derived from the modern documented skeletal collection housed in the School of Legal Medicine at the Faculty of Medicine of the Complutense University of Madrid (Madrid, Spain) [26]. As of 2023 the collection includes 238 individuals and continues to expand due to the ongoing agreement between the University and the funeral services of the Community of Madrid. However, at the time of data acquisition (2010), this twentieth-century collection potentially included 195 accessible individuals (80 females and 115 males) ranging from 3 to 97 years of age.

During the analysis, only mature individuals with the three elements of the innominate fused were selected and remains displaying pelvic pathologies were excluded from the study. This yielded a study sample comprising of 157 individuals. Within this sample, preservation permitted applying the DSP2 methodology to at least one of the hipbones (left and/or right) of most individuals. However, seven right hipbones and four left ones did not have counterparts to undertake corresponding comparisons, attributable to either poor preservation, or total absence. Thus, the final tally of analyzed sample included 303 coxal bones obtained from 157 individuals.

Demographic information derived from obituary records (age-at-death and biological sex) was not available for all individuals. Hence, accuracy investigation, for which documented sex is critical, was undertaken with a reduced dataset of 140 *os coxae* (74 males [54.4%] and 66 females [45.6%]; Table 1). Within this subset, 136 individuals were fully documented (age-at-death and biological sex), and 4 were partially identified (3 males and 1 female; whose ages were unknown). Regarding sex-wise age distribution of this subset, the female subsample was significantly older than males (♀ 68.82 ± 16.26 years vs. ♂ 57.52 ± 19.47 years; Mann-Whitney U = 1566.5; *p* = 0.001), as is observed within osteological documented collections, attributable to differential life expectancy, amongst other factors.

**Table 1:** Demographic information about the documented sample from the University Complutense of Madrid used for the investigation of accuracy outcomes (*N* = 140)

Age group	Males		Females		Total
	*N*	%	*N*	%	
**< 30 years old**	04	5.4	01	1.5	06
**30–39 years old**	12	16.2	05	7.6	19
**40–49 years old**	11	14.9	04	6.1	16
**50–59 years old**	13	17.6	06	9.1	15
**60–69 years old**	05	6.8	10	15.2	19
**70–79 years old**	14	18.9	21	31.8	35
**80–89 years old**	10	13.5	16	24.2	25
**> 90 years old**	02	2.7	02	3.0	04
**Unknown age**	03	4.1	01	1.5	04
**Total**	74	100.0	66	100.0	140

### Sex estimation

During the examination process, the innominate was separated from the rest of the skeleton to prevent complementary information from affecting the objectivity of the results. Thus, the sex estimation technique was applied blindly by the first author, without knowing any biological information for the individuals analyzed. Additionally, no other information that could affect the study (except for the individual’s code) was available where the material is stored.

The DSP tool, originally created and published to enable non-population specific sex estimation in 2005, utilised a simple spreadsheet program where the researcher could type in the maximum possible number of variables (measurements in mm) out of the recommended ten, with a required minimum of four variables. Based on the input data, the probability of each specimen being male or female was automatically computed, with an equal prior probability for male and female groups (p_Male_ = p_Female_ = 0.5). It is important to highlight that although the program worked with a minimum of four variables, the more variables utilised for analysis, the greater was the likelihood of obtaining a significant probability. In addition, sex was determined only if the posterior probability was ≥ 0.95 threshold, which equates to a risk of error of 0.05 i.e., the maximum required for reliable palaeobiological studies [6]. If these probability and error conditions were not fulfilled, an individual was classified as undetermined. The ten measurements that the method employed were the following:

- PUM - Acetabulo-symphyseal pubic length: *minimum distance from the superior and medial point of the pubic symphysis to the nearest point on the acetabular rim at the level of the lunate surface* [27].
- SPU - Cotylo- pubic width: *pubic breadth between the most lateral acetabular point and the medial aspect of the pubis. Measurement is perpendicular to the major axis of the os pubis; arms of the sliding caliper are thus parallel to the plane of the obturator foramen* [28].
- DCOX - Innominate or coxal length: *maximum height of os coxae measured from the inferior border of the os coxae to the most superior portion of the iliac crest. Can be measured with sliding calipers or osteometric board* [27].
- IIMT - Greater sciatic notch height: *distance from the postero-inferior iliac spine (defined as the point of intersection between the auricular surface and the posterior portion of the sciatic notch) to anterior border of the great sciatic notch. Axis of the measurement must be perpendicular to the anterior border. Due to the configuration of hip bone*,* it is easier to use small arms of the sliding caliper* [27].
- ISMM - Ischium post-acetabular length: *distance from the most anterior and inferior point of the ischial tuberosity to the furthest point on the acetabular border* [29].
- SCOX - Iliac or coxal breadth: *distance between the anterior-superior iliac spine and the postero-superior iliac spine* [27].
- SS - Spino-sciatic length: *minimum distance between the antero-inferior iliac spine and the deepest point in the greater sciatic notch* [28].
- SA - Spino-auricular length: *distance between the antero-inferior iliac spine and the auricular point. Auricular point is defined as the intersection of the arcuate line with the auricular surface* [28].
- SIS - Cotylo-sciatic breadth: *distance between the lateral border of the acetabulum and the midpoint of the anterior portion of the great sciatic notch. Fixed arm of the sliding caliper is parallel to the acetabular plane* [27].
- VEAC - Vertical acetabular diameter: *maximum vertical diameter of the acetabulum*,* measured on the acetabular rim*,* as a prolongation of the longitudinal axis of the ischium* [27].

The ten variables are displayed in decreasing order of discriminant interest. The authors of these pivotal studies [10, 11] recommend the first eight variables for sex estimation. The last two dimensions (SIS and VEAC) should mainly be reserved for incomplete bones. All the variables were measured with a standard non-digital sliding caliper and a spreading caliper as proposed by Murail and colleagues [10].

Following the creation of this spreadsheet in 2005, owing to its lack of feasibility, a new scoring software called DSP2 was created in 2017 [11]. Consequently, the analysis within the current study was performed using the DSP2 updated software (http://projets.pacea.u-bordeaux.fr/logiciel/DSP2/dsp2.html), which utilizes Fisher’s linear discriminant analysis (LDA). More detailed information about this mathematical approach is described thoroughly in the original studies [10, 11].

### Statistical analyses

The sex estimation methodology was applied to 157 available mature individuals at that time, using with whom intra-rater reliability, asymmetry and applicability analyses were performed (refer to aims of the study).

In order to address the objectives of this present research, three consecutive sets of measurements were taken in a row from the same individuals: the first set from the left innominate (S1), the second set from the right innominate (S2), and the third set from the left innominate again (S3).

The first and the third sets of measurements (S1 vs. S3) were compared to analyze the intra-observer reliability (*n* = 157). To do this, a two-way random intraclass correlation coefficient (ICC) was computed, due to the nature and characteristics of the data. Due to the data characteristics, absolute agreement ICC type was used and, as reported by Daniel [30], single measures should be employed when intra-observer performance was tested. Obtained ICC values were interpreted according to Koo and Li [31], wherein ICC values lower than 0.5 are indicative of poor reliability, values between 0.5 and 0.75 indicate moderate reliability, values between 0.75 and 0.9 indicate good reliability and values higher than 0.90 indicate excellent reliability. The second and the third sets (S2 vs. S3) were compared to establish potential directional asymmetries (*n* = 157) within the *os coxae*. For this specific analysis, paired t-test and Wilcoxon test was used for normal and non-normal variables, respectively, as and where applicable.

Regarding sexual differences within the DSP2 variables (*n* = 140), student T-test or Mann-Whitney analysis was performed depending on whether the variable follows a normal or a non-normal distribution, respectively.

To estimate accuracy for sexing, S2 (right side) and S3 (left side) were analyzed. Two parameters were computed following the original publication [11]:

- Percentage of sexing *(% sexing*), which constitutes the percentage of specimens whose sex was estimated. To establish the percentage of sexing, a posterior probability equal or superior to 0.95 was considered to be the sex classification threshold.
- Percentage of accuracy (*% accuracy*), which is the percentage of specimens whose sex has been correctly estimated among those calculated.

The *% sexing* parameter is different to *% applicability as* the former considers the percentage of specimens that were classified as male or female (with a posterior probability ≥ 0.95, leaving the undetermined ones out), and the latter takes into account the percentage of specimens where specific variables could be measured based on the individual preservation.

All tests were undertaken using SPSS 29.0. For all statistical assessments, a *p*-value lower than 0.05 was considered statistically significant.

## Results

Based on the values mentioned in the Methods Sect. [31], excellent reliability was achieved during intra-observer analysis for 8 out of 10 DSP variables, with good reliability for PUM and moderate reliability for IIMT (Table 2). Scatterplots of S1 vs. S3 for the variables PUM and IIMT have been displayed as Supporting Information (Supplementary Images 1 and 2) to provide a visual complement to the obtained reliability results.

**Table 2:** Intraclass correlation coefficient (ICC) for intra-observer error analysis for the 10 variables of DSP methodology (*N* = 157) (results obtained on comparing the sets 1 and 3 (S1 vs. S3))

Variable	ICC	95% IC	*p* - value
**PUM**	0.885	0.806–0.928	**< 0.001**
**SPU**	0.949	0.928–0.964	**< 0.001**
**DCOX**	0.970	0.953–0.980	**< 0.001**
**IIMT**	0.659	0.167–0.838	**< 0.001**
**ISMM**	0.969	0.956–0.978	**< 0.001**
**SCOX**	0.942	0.969–0.984	**< 0.001**
**SS**	0.975	0.962–0.983	**< 0.001**
**SA**	0.929	0.902–0.949	**< 0.001**
**SIS**	0.972	0.960–0.980	**< 0.001**
**VEAC**	0.953	0.935–0.966	**< 0.001**

*PUM: Acetabulo-symphyseal pubic length; SPU: Cotylo- pubic width; DCOX: Innominate or coxal length; IIMT: Greater sciatic notch height; ISMM: Ischium post-acetabular length; SCOX: Iliac or coxal breadth; SS: Spino-sciatic length¸SA: Spino-auricular length; SIS: Cotylo-sciatic breadth; VEAC: Vertical acetabular diameter. Significant results are marked in bold*

Regarding potential directional asymmetries, results are displayed in Table 3. Significant differences were observed between the two sides in four variables (DCOX, IIMT, ISMM and SA), while no significant differences between left and right *os coxae* were achieved with the rest of the DSP variables. In the case of DCOX, IIMT and SA, measurements for the left os coxa were significantly higher than right ones, while the opposite was observed for ISMM.

**Table 3:** Directional asymmetry analyses of the DSP2 variables (comparing sets 2 and 3 (S2 vs. S3)) (*N* = 157)

Variable	S2 (right)	S3 (left)	T-test	Fd	Wilcoxon Z	*p* - value
**PUM**	70.59	70.66	x	x	-0.423	0.672
**SPU**	26.29	26.31	x	x	-0.387	0.699
**DCOX**	202.28	202.89	x	x	-2.151	**0.031**
**IIMT**	43.06	43.83	-2.264	123	x	**0.013**
**ISMM**	106.39	105.42	x	X	-4.764	**< 0.001**
**SCOX**	154.32	154.49	-0.607	110	x	0.273
**SS**	69.82	70.00	-0.940	140	x	0.174
**SA**	74.77	75.80	x	X	-3.011	**0.003**
**SIS**	37.39	37.38	0.043	143	x	0.483
**VEAC**	54.57	54.72	x	x	-0.884	0.377

*PUM: Acetabulo-symphyseal pubic length; SPU: Cotylo- pubic width; DCOX: Innominate or coxal length; IIMT: Greater sciatic notch height; ISMM: Ischium post-acetabular length; SCOX: Iliac or coxal breadth; SS: Spino-sciatic length¸SA: Spino-auricular length; SIS: Cotylo-sciatic breadth; VEAC: Vertical acetabular diameter. Significant results are marked in bold*

A comparison of minimum and maximum values for each of the ten DSP variables obtained in the present study, against those reported by Bruzek et al. [11], indicated that all values are within the range reported in the original study [11] (Supplementary Table 1). The only current value that is not falling within the range variation shared by the original authors in the software platform was VEAC, which exceeded the provided maximum score (69 mm vs. 66.5 mm). Nevertheless, it is important to maintain caution during such a comparison, as the original study did not mention the side associated with these measurements, and to maintain uniformity, comparison measurements within the present study have been obtained by combining the right (S2) and left (S3) values.

Regarding sexual dimorphism of the DSP variables for the right and left sides, descriptive statistics are displayed in Table 4. Overall, higher mean values were found in males compared to females except for PUM and IIMT, where the opposite pattern was observed. On the right side (S2), all the DSP variables were significantly sexually dimorphic barring PUM, while on the left side (S3), neither PUM nor SA mean values were significantly different between males and females. The rest of the variables exhibited significant sexual dimorphism.

**Table 4:** Descriptive statistics (by sex) for the *os coxae* variables in the documented sample (*N* = 140)

Variable	*N* (F)	Mean (F)	SD (F)	Min (F)	Max (F)	*N* (M)	Mean (M)	SD (M)	Min (M)	Max (M)	*p* -value (t-test)	*p* – value (Mann-Whitney U)
PUM (R)	56	71.20	4.775	62	85	63	70.26	3.471	61	78		0.514
SPU (R)	56	23.03	2.339	19	31	63	28.89	2.813	21.4	35	**< 0.001**	
DCOX (R)	55	190.49	8.117	172	210	66	212.03	11.093	175	236		**< 0.001**
IIMT (R)	57	44.71	4.810	33	57.6	58	41.52	4.416	32.6	51	**< 0.001**	
ISMM (R)	57	99.12	4.722	90	113.6	65	112.52	5.730	96.6	125		**< 0.001**
SCOX (R)	49	149.98	6.969	133	168	57	157.79	8.546	136	174	**< 0.001**	
SS (R)	62	65.45	3.754	58	76.4	70	73.45	4.482	62	83		**< 0.001**
SA (R)	62	73.26	5.750	57.6	89.7	68	75.43	5.992	61	90	**0.019**	
SIS (R)	62	35.03	3.295	28	41	71	39.52	3.333	31	46	**< 0.001**	
VEAC (R)	60	51.53	3.008	44	62	70	57.36	3.902	49	69		**< 0.001**
PUM (L)	57	71.07	4.651	61	84	63	70.34	4.151	59	81.6	0.182	
SPU (L)	61	22.84	2.333	19	29.3	67	28.92	2.582	21	37		**< 0.001**
DCOX (L)	60	192.10	9.311	172	216	65	212.45	11.054	176	238		**< 0.001**
IIMT (L)	57	45.60	4.560	36	58.6	63	42.49	4.028	31	51	**< 0.001**	
ISMM (L)	61	98.52	5.113	87.6	112	66	111.35	5.810	95	124		**< 0.001**
SCOX (L)	52	150.85	7.188	133	167	60	157.82	8.825	136	181	**< 0.001**	
SS (L)	64	65.81	3.633	57.7	73.7	69	73.36	4.593	64	85		**< 0.001**
SA (L)	64	74.95	6.106	62	92	69	76.54	5.771	65.6	92	0.063	
SIS (L)	65	35.11	3.289	28	42	71	39.32	3.287	31.6	46.4	**< 0.001**	
VEAC (L)	65	51.45	2.657	45	58	71	57.79	3.944	48.8	67		**< 0.001**

*PUM: Acetabulo-symphyseal pubic length; SPU: Cotylo- pubic width; DCOX: Innominate or coxal length; IIMT: Greater sciatic notch height; ISMM: Ischium post-acetabular length; SCOX: Iliac or coxal breadth; SS: Spino-sciatic length¸SA: Spino-auricular length; SIS: Cotylo-sciatic breadth; VEAC: Vertical acetabular diameter. (F): female subsample; (M): male subsample; (R): right innominate (set 2); (L): left innominate (set 3). Significant results are marked in bold*

According to sample preservation, % applicability for every single DSP variable in a sex-pooled sample was higher than 85% in all cases, except for the variable SCOX, where the values were around 80% on both sides (Table 5). Similar results were achieved in males and females separately, with the exception of the right male subsample for IIMT, where the percentage was 81.69%. In the combined sample, larger applicability values were achieved on the left side in all cases, with the exceptions of PUM, SS and SIS. In the sex-specific samples, some variables achieved right predominance and others demonstrated a left predominance, with no consistent pattern. Besides, some of these side differences coincided in males and females separately (PUM, SPU, ISMM, SCOX, SS, VEAC), whereas others did not (DCOX, IIMT, SA, SIS). Regarding applicability for sexing between the two sexes, higher percentages were found in females for most of the DSP variables in the right subsample, except for DCOX, SCOX and VEAC. In the case of SIS, both sexes achieved 100%. On the left sample, however, all female features achieved better results than male ones, with the exception of SCOX.

**Table 5:** Percentage of applicability of the individual variables of DSP2 on the studied sample

	Males (%)		Females (%)		Total (%)	
	Right	Left	Right	Left	Right	Left
**PUM**	88.73	87.50	90.32	87.69	89.33	88.24
**SPU**	88.73	93.06	90.32	93.85	88.67	93.46
**DCOX**	92.96	90.28	88.71	92.31	90.00	90.85
**IIMT**	81.69	87.50	91.94	87.69	86.67	88.24
**ISMM**	91.55	91.67	91.94	93.85	92.00	92.16
**SCOX**	80.28	83.33	79.03	80.00	79.33	81.70
**SS**	98.59	95.83	100.00	98.46	98.00	97.74
**SA**	95.77	95.83	100.00	98.46	96.67	97.39
**SIS**	100.00	98.61	100.00	100.00	100.00	97.39
**VEAC**	98.59	98.61	96.77	100.00	98.00	98.69

*PUM: Acetabulo-symphyseal pubic length; SPU: Cotylo- pubic width; DCOX: Innominate or coxal length; IIMT: Greater sciatic notch height; ISMM: Ischium post-acetabular length; SCOX: Iliac or coxal breadth; SS: Spino-sciatic length¸SA: Spino-auricular length; SIS: Cotylo-sciatic breadth; VEAC: Vertical acetabular diameter. Global dataset (* *n* *= 157) was used for these calculations in the pooled sample while the documented sample (* *n* *= 140) was used to perform the sex-specific calculations. Applicability refers to the number of individuals out of the total where the specific variable could be measured due to preservation*

Concerning the accuracy of right (S2) and left (S3) datasets, general outcomes showed that accuracy percentages were high, being > 94% regardless of the number of variables used (Table 6). Similar values (around 97%) were reached with all variables, the first 8, or the most accurate 4 variables. In fact, even with the worst 4 variables, the accuracy % reached 94.74% and 94.55% for left and right datasets, respectively. Thus, it appears that having the measurements of the first 4 variables is enough to obtain the highest accuracy possible within this pooled sample. However, for the percentage of sexing, the results were different: while similar values were achieved with 10 or the first 8 variables, the values slightly decreased with the best 4 variables and reduced to half with the worst 4 variables. These results were similar when sexes were analyzed separately (Table 6). Thus, accuracy values were always higher than 90% in males and higher than 95% in females, meanwhile sexing percentages decreased when the number and quality of variables decreased as well. However, if the sexes are compared, the females’ results are always higher than the males’ ones, both for sexing and accuracy percentages. The singular exception to this observation is the sexing percentage with the four worst variables on both sides, where the number of estimated males is superior to the estimated females. Lastly, considering sides in the pooled sample, the left dataset achieved better accuracy results in all cases. The same occurred in most of the cases regarding sexing percentages. When sexes are considered in isolation, no clear side pattern was identified, as better results were achieved for left and right datasets in different variable-based categories.

**Table 6:** Sexing accuracy results with various combinations of variables within the documented sample (*N* = 140)

	Undetermined / *N* (% sexing) (F)	Number of errors / determined *N* (% accuracy) (F)	Undetermined / *N* (% sexing) (M)	Number of errors / determined *N* (% accuracy) (M)	Undetermined / *N* (% sexing) (Total)	Number of errors / determined *N* (% accuracy) (Total)
All available variables (L)	1 / 65 (98.46)	0 / 64 (100)	11 / 72 (84.72)	2 / 61 (96.72)	12 / 137 (91.24)	2 / 125 (98.40)
All available variables (R)	3 / 62 (95.16)	0 / 59 (100)	9 / 71 (87.32)	3 / 62 (95.16)	12 / 133 (90.98)	3 / 121 (97.52)
10 variables (L)	0 / 45 (100)	0 / 45 (100)	6 / 48 (87.50)	1 / 42 (97.62)	6 / 93 (93.55)	1 / 87 (98.85)
10 variables (R)	0 / 44 (100)	0 / 44 (100)	5 / 46 (89.13)	2 / 41 (95.12)	5 / 90 (94.44)	2 / 85 (97.65)
First 8 variables (L)	0 / 45 (100)	0 / 45 (100)	6 / 49 (87.76)	1 / 43 (97.67)	6 / 94 (93.62)	1 / 88 (98.86)
First 8 variables (R)	0 / 44 (100)	0 / 44 (100)	5 / 46 (89.13)	2 / 41 (95.12)	5 / 90 (94.44)	2 / 85 (97.65)
Best 4 variables (L)	2 / 47 (95.83)	0 / 45 (100)	9 / 55 (83.64)	2 / 46 (95.65)	11 / 103 (89.32)	2 / 92 (97.83)
Best 4 variables (R)	0 / 47 (100)	0 / 47 (100)	12 / 51 (76.47)	2 / 39 (94.87)	12 / 98 (87.76)	2 / 86 (97.67)
Worst 4 variables (L)	40 / 64 (37.50	0 / 24 (100)	34 / 67 (49.25)	3 / 33 (90.91)	74 / 131 (45.51)	3 / 57 (94.74)
Worst 4 variables (R)	40 / 60 (33.33)	1 / 20 (95.00)	32 / 67 (52.24)	2 / 35 (94.29)	72 / 127 (43.31)	3 / 55 (94.55)

*% sexing means the percentage of specimens whose sex has been determined (p ≥ 0.95) while % accuracy means the percentage of specimens whose sex has been correctly determined among those determined. Results are given for both sides separately and*,* regarding biological sex*,* for the pooled sample and each sex separately. First 8 variables: without SIS and VEAC. Best 4 variables: DCOX*,* PUM*,* SPU and IIMT. Worst 4 variables: SIS*,* VEAC*,* SA and SS. (L): left. (R): right. (F): female. (M): male*

With the aim of investigating the relevance of the posterior probability threshold for accuracy estimations, previous outcomes were compared against sexing accuracy obtained by decreasing the posterior probability from 95% (as original authors advised) to 85% (Table 7). The percentage of sexing increased in all cases, with a significant increase observed in males. Nevertheless, decreasing the posterior probability by 10% also reduced the percentage of accuracy in most cases, with few right-side exceptions where similar results were obtained.

**Table 7:** Results with various combinations of variables based on the posterior probability (95% vs. 85%) within the documented sample (*N* = 140)

	Posterior probability ≥ 0.95		Posterior probability ≥ 0.85		Results				*N* (total)
	% sexing	% accuracy	% sexing	% accuracy	Sexing (M)	Sexing (F)	Number of new errors (M)	Number of new errors (F)	
All available variables (L)	91.24	98.40	94.90	97.69	+ 5 (11)	+ 0 (1)	+ 1 (5)	+ 0 (0)	137
All available variables (R)	90.98	97.52	96.24	97.66	+ 7 (9)	+ 0 (3)	+ 0 (7)	+ 0 (0)	133
10 variables (L)	93.55	98.85	97.85	97.80	+ 4 (6)	+ 0 (0)	+ 1 (4)	+ 0 (0)	93
10 variables (R)	94.44	97.65	97.78	97.73	+ 3 (5)	+ 0 (0)	+ 0 (3)	+ 0 (0)	90
First 8 variables (L)	93.62	98.86	97.87	97.83	+ 4 (6)	+ 0 (0)	+ 1 (4)	+ 0 (0)	94
First 8 variables (R)	94.44	97.65	97.78	97.73	+ 3 (5)	+ 0 (0)	+ 0 (3)	+ 0 (0)	90
Best 4 variables (L)	89.32	97.83	95.15	96.94	+ 4 (9)	+ 2 (2)	+ 1 (4)	+ 0 (2)	103
Best 4 variables (R)	87.76	97.67	96.94	96.84	+ 9 (12)	+ 0 (0)	+ 1 (9)	+ 0 (0)	98
Worst 4 variables (L)	45.51	94.74	76.34	93.00	+ 17 (34)	+ 26 (40)	+ 3 (17)	+ 1 (26)	131
Worst 4 variables (R)	43.31	94.55	66.14	92.86	+ 19 (40)	+ 10 (32)	+ 3 (19)	+ 0 (10)	127

*% sexing means the percentage of specimens whose sex has been determined (p ≥ 0.95) while % accuracy means the percentage of specimens whose sex has been correctly determined among those determined. Results are given for both sides separately and*,* regarding biological sex*,* for the pooled sample and each sex separately. First 8 variables: without SIS and VEAC. Best 4 variables: DCOX*,* PUM*,* SPU and IIMT. Worst 4 variables: SIS*,* VEAC*,* SA and SS. L: left. R: right. F: female. M: male*

*In the “Sexing” columns belonging to “Results”: 1) Plain numbers means the number of individuals who were determined with the 0.85 threshold but not with the 0.95 one. The number between parentheses shows the total number of undetermined individuals with the 0.95 threshold. In the “Number of new errors” columns belonging to “Results”: 1) Plain numbers means the number of new committed errors in sex estimation when using the 0.85 threshold instead of the 0.95 one. The number between parentheses shows the number of individuals who were determined with the 0.85 threshold but not with the 0.95 one*

## Discussion

Sex estimation is one of the first procedures towards human identification within medicolegal and forensic contexts. Accurate sex estimation can also contribute to providing important insights into population history and migration patterns. Therefore, this step is an essential tool for forensic anthropologists and archaeologists, and it is crucial for a comprehensive understanding of human evolution, biology, and health. Numerous research has previously been published on metric sex estimation for diverse Spanish samples, using different skeletal elements: the skull [32], the dentition [33, 34], the clavicle [35–37], the sternum [38], the ribs [39, 40], the vertebrae [41–43], the sacrum [44], the radius [45], the carpals [46], the femur [47, 48], the patella [37, 49], the tibia [50, 51], the talus [45], the navicular [52] or the metacarpals [53]. However, despite its direct association with parturition and proven accuracy for being the most dimorphic human bone, specific research on sex estimation using hipbone measurements is currently limited for this biogeographical population [47, 54]. In this respect, to the best of our knowledge, DSP2 has not been tested in any Spanish sample, so comparisons along this line are not feasible.

### Reliability

Overall, the current study shows excellent results for intra-rater reliability analysis, coinciding with previous literature for pelvic measurements [15, 55, 56]. However, good and moderate results were achieved with PUM and IIMT, respectively, yielding lower ICC values compared to the ones reported by de Almeida in a Brazilian sample [15]. The comparatively lower values for sciatic greater notch (IIMT) have already been reported during previous reliability analyses [15, 55], and with CT-based studies [19, 20]. These results are likely due to different factors such as the greater difficulty associated with identifying the anatomical landmarks and placing the sliding caliper in the correct position, which requires the user to visually check a right angle between the postero-inferior iliac spine and the anterior border of the greater sciatic notch [27]. In addition, it is worth highlighting that these outcomes contrast with the fact that both features, PUM and IIMT, belong to the first four DSP variables, considered to have the highest discrimination power [10]. Consequently, special care should be taken when using these specific variables, and better descriptions and/or images could be provided in future DSP updates.

### Applicability

The current percentage applicability for SCOX was lower in comparison to other variables. This outcome corroborated previous literature results [11, 13, 14]. However, those authors reported similar lower values for PUM, contrasting to our findings. Furthermore, overall better female preservation (% applicability: possibility of taking the measurements) compared to males was found (except SCOX), contrasting to the higher gracility and associated fragility of skeleton of women. This could be linked to their belonging to a documented collection from modern cemeteries, with different taphonomic processes or artificial barriers such as coffin protection, slowing down the natural human decomposition. In addition, the female mean age was significantly higher than the male’s in this study sample, rendering their skeletons more prone to be vulnerable and fragmentary, so the current results regarding applicability should be explained by other different factors. The SCOX exception, on the other hand, may be due to both postero-superior and antero-superior spines involved in the measurement. These landmarks are likely to be more robust, and so preservable in males due to anatomical muscular attachments.

### Sexing and accuracy percentages

#### Overall results

Current results show that, whereas the sexing % decreased progressively when the number of variables or their discrimination power was reduced, the accuracy % remained high irrespective of the combination of employed variables. A comprehensive comparison to previously reported similar literature is shown in Table 8. Overall, results from all ten, or the first eight variables are almost identical across the table, so it appears that when the first eight variables are available for analysis, incorporating the remaining two (SIS and VEAC) does not render better outcomes. As advocated by Murail et al. and Bruzek et al. [10, 11], the last two features are useful within degraded and/or fragmentary contexts, where other more accurate variables are non-viable. In such scenarios, these variables with relatively lower accuracy can aid in identification by helping achieve the minimum four-variable requirement mandated by the DSP software.

**Table 8:** Comparison of results across literature and the present study when applying DSP methodology in pooled samples belonging to diverse populations based on the number of variables used

TOTAL			10 variables		8 variables		“Best” 4 variables		“Worst” 4 variables	
Reference	Geographical origin	*N*	% sexing	% accuracy	% sexing	% accuracy	% sexing	% accuracy	% sexing	% accuracy
Murail et al., 2005 [10]	European (UK, France, Portugal)	454	-	-	95.9	100	92.8	100	69.7	98.3
Murail et al., 2005 [10]	African American ^1^	329	-	-	92	98.6	86.9	98.6	66.1	98.6
Murail et al., 2005 [10]	European American ^1^	311	-	-	93	100	89.5	99.6	65	97.4
Murail et al., 2005 [10]	European + North American	1094	-	-	99.7	99.3	86.9	99.7	76	99.6
Murail et al., 2005 [10]	Thailand ^2^	198	-	-	94.1	100	90.5	100	75.5	100
Murail et al., 2005 [10]	Lithuania ^2^	220	-	-	94.4	100	91.7	100	71.6	98.7
Murail et al., 2005 [10]	South Africa – Zulu ^2^	306	-	-	88.7	98.8	84.6	98.8	66.2	99
Murail et al., 2005 [10]	South Africa – Soto ^2^	110	-	-	86	100	84.4	100	63	100
Murail et al., 2005 [10]	South Africa – Afrikaner ^2^	112	-	-	95.1	100	88.8	100	70.8	100
Murail et al., 2005 [10]	Worldwide	2040	90.71	99.63	90.76	99.63	86.69	99.61	40.23	98.75
Sánchez-Mejorada et al., 2011 [14]	Mexico	250	89.2	100	-	-	-	-	-	-
Chapman et al., 2014 [18]	Belgium	39	97.4	100	97.44	100	89.74	100	53.85	90.48
Bruzek et al., 2017 [11]	France	160	89.93	100	90.07	100	83.22	99.19	45.57	100
Bruzek et al., 2017 [11]	Portugal	232	89.64	100	89.69	100	86.54	98.33	44.78	99.03
Bruzek et al., 2017 [11]	United Kingdom	62	86.54	100	86.54	100	80.7	100	32.79	100
Bruzek et al., 2017 [11]	Lithuania	220	95.39	100	95.41	100	92.73	100	39.91	100
Bruzek et al., 2017 [11]	South Africa - Zulu	306	88.44	99.23	88.78	99.23	84.85	100	33.44	98.04
Bruzek et al., 2017 [11]	South Africa - Soto	110	85.44	100	85.44	100	80.73	100	43.4	95.65
Bruzek et al., 2017 [11]	South Africa - Afrikaner	112	93.62	100	94	100	89.72	100	43.27	100
Bruzek et al., 2017 [11]	European American	112	93.62	100	93.68	100	90.48	100	42.45	100
Bruzek et al., 2017 [11]	African American	113	90.2	98.91	90.2	98.91	87.04	100	40.18	97.78
Bruzek et al., 2017 [11]	European American	199	88.24	100	89.01	100	84.02	99.39	49.22	97.89
Bruzek et al., 2017 [11]	African American	216	91.71	98.4	91.39	98.43	89.57	98.41	45.02	96.84
Bruzek et al., 2017 [11]	Thailand	198	94.62	100	94.62	100	91.1	100	38.14	100
Bruzek et al., 2017 [11]	North America	120	93.46	99	93.58	99.02	87.27	98.96	50.86	94.92
Bruzek et al., 2017 [11]	Switzerland	503	94.74	96.03	94.78	96.06	90.91	96.88	55.25	98.06
Bruzek et al., 2017 [11]	Worldwide	2040	90.84	99.65	90.98	99.65	87.17	99.53	41.49	98.67
Quatrehomme et al., 2017 [12]	France	100	94.83	100	94.92	100	76.92	100	52.87	100
Salles Machado et al., 2018 [17]	Brazil	103	85.43	88.34	-	-	82.52	90.29	60.19	93.20
Rodriguez Paz et al., 2019 [20]	Denmark	116	93.9	100	93.1	100	81.9	100	49.7	98.2
Kranioti et al., 2019 [13]	Greece	133	88.00	97.43	-	-	-	-	-	-
De Almeida et al., 2020 [15]	Brazil	301	94.00	99.3	94.7	99.3	90.7	100	61.0	98.9
Rodrigo de Oliveira Lopes et al., 2023 [16]	Brazil	128	-	-	-	-	71.09	92.97	-	-
Oh et al., 2023 [24]	South Korea	29	86.2	86.2	89.66	86.2	85.00	80	31.3	31.03
**Current study (right)**	**Spain**	**140**	**94.44**	**97.65**	**94.44**	**97.65**	**87.76**	**97.67**	**43.31**	**94.55**
**Current study (left)**	**Spain**	**140**	**93.55**	**98.85**	**93.62**	**98.86**	**89.32**	**97.83**	**45.51**	**94.74**

^*1*^ *Results reported from Murail et al. testing the European model (reference) on North American target samples*

^*2*^*Results reported from Murail et al. testing the European and North American model (reference) on different targets: Asian (Thailand)*,* African (South Africa) and European (Lithuania) samples*

*% sexing means the percentage of specimens whose sex has been determined (* *p* *≥ 0.95) while % accuracy means the percentage of specimens whose sex has been correctly determined among those determined*

Sexing % values obtained with all ten variables in the present study were 94.44% and 93.55% for the right and left halves, respectively, which is slightly lower than the 97.40% reported by Chapman et al. [18], the maximum value found in the literature, and higher than the 85.43% reported by Salles Machado et al. [17], the minimum value achieved across referenced studies (Table 8). Regarding % accuracy, the present study achieved 97.65 and 98.85 (right and left, respectively), within the range from the minimum 86.20% [24] to the maximum, 100.0% [11, 12, 14, 18–20]. A comparative analysis (Table 8) indicates that general % accuracy results are consistently high in literature, with most of the authors reporting values higher than 95% with only two exceptions: a Brazilian miscegenated population [17] and a very small sample from South Korea [24]. In fact, several authors found no cases of misclassification during their sex estimation by DSP [11, 12, 14, 18–20]. In the current study, this sort of success rate was achieved only in females.

The present results of % accuracy with the best 4 variables were 97.67% and 97.83% for right and left, respectively. According to the previous studies (Table 8), the reported results ranged from 80% [24] to 100% [10–12, 15, 18, 20]. However, the lowest value (80%) came from an already mentioned small sample from Korea comprising of only 29 individuals. The second lowest result generated with a larger sample size (*n* = 103) was reported as 90.29% [17]. In this specific case [17], the results for % accuracy should be taken with caution since some reported values are unexpected and difficult to justify; i.e. they rendered 88.34%, 90.29% and 93.20% using 10 variables, best 4 variables and worst 4 variables, respectively. Continuing with best 4 variables results, all other studies yielded accuracy percentages higher than 90%. However, outcomes of % sexing are quite different: while the present study’s ones were 87.76% and 89.32% for right and left sides, respectively, previously literature results ranged from 71.09% [16] to 92.8% for a specific European subsample [10].

Results of the worst 4 variables achieved good accuracy % outcomes in the present study: 94.55% and 94.74% for the right and left sides, respectively. Regarding previous studies, if the Korean small sample study is not considered due to its smaller sample size, the results ranged between 90.48% [18] and 100% [10–12]. In the case of the % sexing, the scenario is different. The minimum outcome was reported in a British subsample published by the original authors [11] as 32.79%, while the maximum was also found within the original publication [10] as 76%. Murail and colleagues’ original publication reported a sexing % of 40.23% in the worldwide sample (*n* = 2040) but, surprisingly, higher outcomes were reported in its different subsamples (even when 7 out of 9 of them were testing reference models on geographically different target samples), ranging from 63 to 76%. The rest of the revised literature, including the updating of DSP as DSP2 [11], reported % sexing between 32.79% [11] and 61.0% [15]. A scientific explanation for Murail’s differential findings is currently lacking.

One of the drawbacks of DSP compared with other discriminant analysis is the high number of undetermined cases that the software generates, especially when a reduced number of variables are considered. These results arise of the original authors decision about employing a 0.95 threshold instead of the 0.50 value. This settlement, however, guarantees very high levels of accuracy, with 100% or close values with a combination of the most discriminant variables. Interestingly, even taking into account just the four variables with relatively lower discrimination power (SS, SA, SIS and VEAC), accuracy percentages were lower than 94.0% in just 3 out of 32 revised literature outcomes, including the Korean one with a sample size of 29 individuals. Nevertheless, as other authors have suggested [20], the poorer results with the worst 4 combination of variables (or with a reduced number of variables) need to be interpreted with caution and complemented with non-metric methodologies.

In the present study, the posterior probability threshold was decreased from 95 to 85% to assess its potential impact on % sexing and % accuracy. This resulted in an increase in the % sexing in all cases in exchange of reduced % accuracy in most of the cases. Since the current female outcomes were comparably better than males, most of the changes were associated with sexing in males: improvement by increasing the sexing rate but gaining imprecision by incorporating errors. While this reduced accuracy still has acceptable values, the reduction in posterior probability is not recommended within future forensic practice from a reliability point of view, as a decrease in the number of indeterminate bones does not necessarily make up for decreased reliability and accuracy. Given the medico-legal and forensic contexts of interest here, an unreliable sex estimation approach can be more problematic than an unapplicable one [57, 58]. Furthermore, the reduction in posterior probability here, by and large, also resulted in a decrease in % accuracy, negating the need for this reduction. Moreover, even with a 95% posterior probability, % accuracy obtained in the present study is high (even with the worst four variables), demonstrating an excellent applicability for the DSP2 approach when it comes to sex estimation.

In relation to misclassification cases, some documentary or human error should not be completely ruled out in the osteological collection itself, since worldwide researchers and curators work with them all the time and misplacing skeletal elements or typographical errors could be contributing factors [18, 59].

#### Side differences

Most of the authors who have analyzed DSP performance in diverse populations have used the left side throughout to be consistent [13, 15, 17, 18, 20]. However, some researchers used left and right hip bones in their studies, combining both sides without analyzing for potential differences [14, 19]. Whereas other studies only partially examined them [12, 16]. The present study has found higher values in all left cases compared to the right-side results, apart from ISMM, with opposite results, and a quite identical mean value in SIS between sides. However, these differences were only statistically significant in DCOX, IIMT, ISMM and SA. This finding could not be corroborated as no similar results have been reported previously, warranting further investigation into the causality of this anomaly. In fact, other authors concluded that the DSP values from the right and left coxal bones were comparable [12], even suggesting that single measurements may be substituted in cases of non availability of both halves of the pelvis. This absence of asymmetry agrees with previous results from a CT-based investigation which utilised different pelvic measurements [60].

Overall, current results did not indicate any clear side-based results regarding the % sexing in different number of variables sets. However, left side achieved better % accuracy compared to the right in all cases (Table 6). In females, similar results were found. No differences were displayed in most of the cases of % accuracy in females as left and right sides both achieved 100%. In males, on the other hand, right side displayed higher % sexing in all cases except for the best 4 variables, while left side achieved better % accuracy than right side with one exception: the worst 4 variables case. The only previous investigation to compare those results was from Rodrigo de Oliveira Lopes et al. [16], who reported accuracy comparison for the best 4 variables (PUM, SPU, DCOX, IIMT). These authors found higher % sexing and % accuracy in the left side in females and in the right side in males, i.e., contrasting findings in comparison to the present study (Table 6). Accuracy % results in females were not comparable because both left and right sides achieved 100% in the present study.

#### Sexual differences

Neither PUM nor SA mean metric values were found to have significant differences between sexes in the left side coinciding with Salles Machado et al. [17]. In the right side, PUM alone did not exhibit significant sex differences, a result unreported in the literature. Other authors describe non-significant differences between males and females in SA [11, 13–15, 19] and in the combination of PUM, SCOX and SA [12]. These PUM results highly contrast with the fact that this variable has the highest discrimination power across the analyzed ten variables according to the DSP creators [10], data which contrasts with Kranioti et al., who described the four most discriminant variables as ISMM, SPU, DCOX and VEAC in a Greek sample [13].

Tables 9 and 10 display the comparison of sex-specific percentages of sexing and accuracy depending on the number of DSP variables used across scientific literature. In the female comparative analysis (Table 9), the present study achieved 100% in most of the cases of % sexing in the first three sets (10, 8 and best 4 variables), exceeding the reported outcomes in other samples. Regarding % accuracy, no misclassification cases were reported here, agreeing with the ones rendered in Mexican [14], French [12, 19] and Brazilian [15] samples. However, the low % sexing exhibited in French (41.66%) compared to the rest of analyzed samples using the best four variables, ranging from 85.2 to 100%, is hard to explain, and is more similar to results from the set of the worst 4 variables. Since some of the values reported by Quatrehomme et al. [12] are internally inconsistent within their own tables, these results could have been caused by formatting typos and should be compared with caution. In the case of the worst 4 variables, present results are slightly lower than other studies [11, 12, 17]. It is remarkable that the high percentage achieved in this case by de Almeida and colleagues [15] in a Brazilian sample (81.2%), is more similar to the results of 10, 8 or best 4 variables in other geographical regions.

**Table 9:** Comparison of literature results with current ones when applying DSP methodology in female samples belonging to diverse populations based on the number of variables used

FEMALES			10 variables		8 variables		“Best” 4 variables		“Worst” 4 variables	
Reference	Geographical origin	*N*	% sexing	% accuracy	% sexing	% accuracy	% sexing	% accuracy	% sexing	% accuracy
Sánchez-Mejorada et al., 2011 [14]	Mexico	118	98.31	100	-	-	-	-	-	-
Mestekova et al., 2015 [19]	France	54	97.2	100	-	-	85.2	100	-	-
Bruzek et al., 2017 [11]	Worldwide	1023	90.96	99.29	91.21	99.30	87.55	99.53	42.54	98.82
Quatrehomme et al., 2017 [12]	France	-	85.71	100	94.91	100	41.66	100	40.48	100
Salles Machado et al., 2018 [17]	Brazil	50	98.05	86.00	-	-	92.00	88.00	38.00	82.00
Rodriguez Paz et al., 2019 [20]	Denmark	67	97.0	-	-	-	93.3	-	-	-
De Almeida et al., 2020 [15]	Brazil	136	94.4	100	95.8	100	88.7	100	81.2	99.0
Rodrigo de Oliveira Lopes et al., 2023 [16]	Brazil	50	-	-	-	-	82.0	96.0	-	-
**Current study (right)**	**Spain**	**66**	**100**	**100**	**100**	**100**	**100**	**100**	**33.33**	**95.0**
**Current study (left)**	**Spain**	**66**	**100**	**100**	**100**	**100**	**95.83**	**100**	**37.50**	**100**

*% sexing means the percentage of specimens whose sex has been determined (* *p* *≥ 0.95) while % accuracy means the percentage of specimens whose sex has been correctly determined among those determined*

**Table 10:** Comparison of literature results with current ones when applying DSP methodology in male samples belonging to diverse populations based on the number of variables used

MALES			10 variables		8 variables		“Best” 4 variables		“Worst” 4 variables	
Reference	Geographical origin	*N*	% sexing	% accuracy	% sexing	% accuracy	% sexing	% accuracy	% sexing	% accuracy
Sánchez-Mejorada et al., 2011 [14]	Mexico	132	81.06	100	-	-	-	-	-	-
Mestekova et al., 2015 [19]	France	52	92.3	100	-	-	96.2	100	-	-
Bruzek et al., 2017 [11]	Worldwide	1017	90.72	100	90.75	100	86.79	99.53	40.44	98.51
Quatrehomme et al., 2017 [12]	France	-	100	100	100	100	97.56	100	64.44	100
Salles Machado et al., 2018 [17]	Brazil	53	89.32	90.57	-	-	73.58	73.58	79.24	100
Rodriguez Paz et al., 2019 [20]	Denmark	49	89.7	-	-	-	86.3	-	-	-
De Almeida et al., 2020 [15]	Brazil	165	93.7	98.6	93.7	98.6	92.4	100	43.5	98.5
Rodrigo de Oliveira Lopes et al., 2023 [16]	Brazil	78	-	-	-	-	64.10	91.03	-	-
**Current study (right)**	**Spain**	**74**	**89.13**	**95.12**	**89.13**	**95.12**	**76.47**	**94.87**	**52.24**	**94.29**
**Current study (left)**	**Spain**	**74**	**87.5**	**97.62**	**87.76**	**97.67**	**83.64**	**95.65**	**49.25**	**90.91**

*% sexing means the percentage of specimens whose sex has been determined (* *p* *≥ 0.95) while % accuracy means the percentage of specimens whose sex has been correctly determined among those determined*

On the other hand, male-specific analysis is showed in Table 10. Regarding the 10-variable and 4-best cases, where more references were reported, current study % sexing are lower than the previous publications, except in Mexican [14] and Brazilian populations [16, 17]. For % accuracy, similar overall lower accuracy was achieved in the current study when compared with other publications with the exception of the Brazilian population [16, 17]. As mentioned previously, Salles Machado et al. [17] results are anomalous and should be considered cautiously; e.g. % accuracy using the worst 4 variables (100%) is higher than the value resulted from using 10 variables (90.57%). With regards to the worst 4 variables results, present % sexing results are higher than Northeastern Brazilian [15] and the worldwide sample utilised by Bruzek and colleagues [11] but lower than the outcomes achieved for France [12] and Southeast Brazil populations [17]. It is worthy to highlight the high percentage achieved by Salles Machado et al. [17] in a Brazilian sample (79.24%), comparable to the results of 10, 8 or best 4 variables in other geographical regions. The existence of an outlier outcome had been found and previously commented in Brazilian females, but in a different sample [15]. These differences could be explained by the great geographical extent and human diversity of this country, likely existing intrapopulation differences between Northeastern [15] and Southeast [17] Brazilian samples. To conclude the comparison, the current % accuracy results for the worst 4 variables in males were lower than all the revised literature ones [11, 12, 15, 17].

Comparing sexes across populations, in the case of the performance of the DSP methodology, both the sexing and accuracy results were better for females than males in most of the cases in the current sample. Higher % sexing, with less undetermined individuals in females, has already been reported in Mexicans [14], Danish [20], Brazilians [16] and the worldwide sample offered by Bruzek et al. [11]. Contrasting results were found for a French population [12]. Furthermore, outcomes in favor of females or males depending on the number of used variables, with no clear sex-specific trend, were found in French [19], Brazilian [15, 17], and Belgians [22]. According to % accuracy, on the other hand, the female predominance compared to males found here agreed with some Brazilian outcomes [15, 16] and were also reported in pre-Columbian mummies [22]. Besides, Bruzek et al. (worldwide sample) found more accurate results in males in the first two subsamples (10 and 8 variables) and coinciding or similar outcomes with the best and worst 4 variables subsamples, respectively [11]. No differences were found in % accuracy between sexes in Mexicans [14], and French [12, 19], where the systematic shared results between sexes were 100%. Contrasting with current findings, Gonzalez et al. examined sexual dimorphism in the great sciatic notch and ischiopubic complex in a Portuguese sample [61], concluding females are misclassified more often than males.

#### Inter-population differences

Current DSP variables values were within the ranges described by the worldwide sample used by the original authors [10, 11] with the exception of VEAC, where the current data was higher than the worldwide-based maximum threshold reported by Bruzek et al. [11]. Some research [62] stated that skeletal sexually dimorphic characters show inter and intra-population variability and other authors stated that pelvic-based sex determination may be impacted by population differences and sample variability [56, 63]. However, other researchers advocate for the opposite, specifically regarding the pelvis [64]. In keeping with this, Bruzek and colleagues [11] stated that the pelvis shows a similar pattern of sexual dimorphism across diverse geographical areas which appeared approximately 100–150 ky ago in early modern humans, defended by previous literature [65–69]. Thus, they offered a worldwide database (software DSP2) of pelvic measurements to any anthropologist who needs to sex skeletal remains, regardless of their geographical origin. This global reference not only comes from samples around the world but also from different ethnical groups (Zulu, Soto, Afrikaner, African American, and European American) and temporal periods (from 18th to late 20th centuries), enhancing their potential applicability in miscellaneous populations. At this respect, it is worthy to stress that when reference models were used to determine the sex of geographically different target populations [10], accuracy results from 97.4 to 100% were displayed (Table 8). However, some large geographical areas were not considered in the reference sample used to calculate the posterior probabilities on DSP2, such as Central or South America. In addition, Africa and Asia were scarcely represented, with just Thai and South African samples analyzed. Although posterior tests on Mexican [14] and Brazilian [15, 16] samples reported comparable percentages of sexing and accuracy, application to miscegenated Brazilian-identified sample [17] yielded lower values compared to published literature (Table 8). Furthermore, similar results arose when DSP2 and population-specific formulae were applied to a Greece sample [13]. However, Kranioti et al. recommended population-specific formulations whenever possible to maximize the outcomes. This existing inter-population variation was also suggested by the application of DSP on Pre-Columbian mummies [22], which garnered lower measurements for the DSP variables when compared to the original reference ranges [11].

## Conclusions and future directions

This study investigated thoroughly the application of DSP2 to a Spanish dry skeletal sample. Percentage of sexing was high when 10, first 8 or 4 best variables were considered and reduced to half when the 4 variables with least discrimination power, called worst 4 variables, were utilized. Regarding % accuracy, however, they were high in all cases, especially in females, regardless of the number of variables used. As the original authors described [10], the “worst” combination of four variables only refers to the decreasing percentage of sexed specimens, while the accuracy remains extremely high. The benefits and utility of the DSP method seem to have driven to consolidate also in bioarchaeological contexts [21–23, 25, 70–72]. However, their actual accuracy on past populations is poorly understood, so further comparison between DSP outcomes on genetic-based documented samples should be implemented in paleontological and archaeological remains.

Despite being restrictive, the suggested posterior probability of 0.95 seems to make the DSP reliable and accurate, rendering it a meaningful tool in forensic practice. Besides, its high flexibility regarding the variables used gives it a special place in forensic anthropology lab routinely working on fragmentary or degraded samples. DSP has also yielded good accuracy results when applied to CT scan images [18–20]. These virtual-based results open the possibility to new population-specific tests, which may be performed in geographical areas hardly examined so far, such as Asia or Africa, where documented collections are scarce or working with skeletal remains is more difficult for international researchers.

## Electronic supplementary material

Below is the link to the electronic supplementary material.

Supplementary Material 1

## Data Availability

N/A.
