# Supplementary material for: Application of DSP2 for biological sex estimation in a Spanish sample: analysis based on sex and side
Source: Int J Legal Med. 2024 Nov 15;139(2):847–62. doi: 10.1007/s00414-024-03358-1 (PMC11850468; doi:10.1007/s00414-024-03358-1)
Supplement: Supplementary file 1 — Supplementary Material 1 [file 414_2024_3358_MOESM1_ESM.docx]

**Supplementary Image 1.** Scatterplot of Sets 1 (X-axis) vs. 3 (Y-axis) for the DSP2 variable PUM (acetabulo-symphyseal pubic length).

**Supplementary Image 2.** Scatterplot of Sets 1 (X-axis) vs. 3 (Y-axis) for the DSP2 variable IIMT (greater sciatic notch height).

**Supplementary Table 1.** Comparison between the minimum and maximum values of the DSP2 variables obtained from the current study and reported within the original study [11].

| Variable | Original sample *(Bruzek et al., 2017) [11]*  [N = 2040] | | Current sample  [N = 157] | |
| --- | --- | --- | --- | --- |
|  | *Minimum* | *Maximum* | *Minimum* | *Maximum* |
| PUM | 56.0 | 87.0 | 59.0 | 85.0 |
| SPU | 17.0 | 38.5 | 19.0 | 37.0 |
| DCOX | 170.0 | 253.0 | 172.0 | 238.0 |
| IIMT | 23.0 | 63.0 | 31.0 | 58.6 |
| ISMM | 86.9 | 131.5 | 87.6 | 125.0 |
| SCOX | 123.0 | 187.0 | 133.0 | 181.0 |
| SS | 52.5 | 91.0 | 57.6 | 85.0 |
| SA | 53.5 | 94.7 | 57.0 | 92.0 |
| SIS | 26.5 | 52.0 | 28.0 | 48.0 |
| VEAC | 42.0 | 66.5 | 44.0 | 69.0 |

*PUM: Acetabulo-symphyseal pubic length; SPU: Cotylo- pubic width; DCOX: Innominate or coxal length; IIMT: Greater sciatic notch height; ISMM: Ischium post-acetabular length; SCOX: Iliac or coxal breadth; SS: Spino-sciatic length; SA: Spino-auricular length; SIS: Cotylo-sciatic breadth; VEAC: Vertical acetabular diameter.*
